# ﻿A new species of the long-tailed wasp genus *Euurobracon* Ashmead (Hymenoptera, Braconidae, Braconinae) from Java, Indonesia, is described and the type species redescribed

**DOI:** 10.3897/zookeys.1116.84593

**Published:** 2022-08-05

**Authors:** Donald L. J. Quicke, Dian Gafar, Kyohei Watanabe, Buntika A. Butcher

**Affiliations:** 1 Integrative Ecology Laboratory, Department of Biology, Faculty of Science Chulalongkorn University, Bangkok 10330, Thailand Chulalongkorn University Bangkok Thailand; 2 Bandung, West Java, Indonesia Bandung West Java Indonesia; 3 Kanagawa Prefectural Museum of Natural History, Iryuda 499, Odawara, Kanagawa 250-0031, Japan Kanagawa Prefectural Museum of Natural History Odawara Japan

**Keywords:** 16S, COI, cytochrome b, DNA sequences, Japan, mitochondrial genes, molecular analysis, Oriental, parasitoid wasp

## Abstract

A new species, *Euurobraconbhaskarai* Quicke, **sp. nov.**, from West Java, Indonesia, is described, illustrated and differentiated from other members of the genus. It is closely related to the type species of the genus, *E.yokahamae* Dalla Torre, 1898, which is known from China, India, Japan, Laos, South Korea and Thailand. *Euurobraconyokahamae* is redescribed and illustrated for comparative purposes. The two species are separable mainly on colouration, but differ markedly based on their mitochondrial gene sequences (cytochrome c oxidase I, cytochrome b and 16S rDNA). The slower-evolving nuclear 28S rDNA and elongation factor 1-alpha did not differentiate *E.bhaskarai***sp. nov.** from *E.yokahamae*, but consistently split *Euurobracon* into two species groups.

## ﻿Introduction

Some of the largest braconid parasitoid wasps belong to the braconine genus *Euurobracon* Ashmead, 1900, which is distributed from the East Palaearctic (China, Japan, Korea), throughout the Oriental region (India, Sri Lanka and South East Asia) and reaches the Australasian region (Papua New Guinea) (Quicke 1989a). Female body length reaches 21 mm and their ovipositors can be up to 18.8 cm long. The genus comprised a total of 14 described species (Li et al. 2016; Yu et al. 2016), of which 11 were included in the revision by Quicke (1989a) and three more from China that were added by Li et al. (2016). Despite its wide distribution, only four species have been recorded from Indonesia: *E.cephalotes* (Smith, 1858) (Java, Sumatra and N. Mollucas), *E.forticornis* (Cameron, 1905) (Sumatra and N. Mollucas), *E.impossibilis* (Dalla Torre, 1898) (N. Mollucas), and *E.denticephalus* Quicke, 1989 (Irian Jaya). However, *E.interstitialis* Quicke, 1989 which is known from Sarawak and Sabah on Borneo Island must surely also occur in that country. Of these, only *E.cephalotes* has previously been recorded from Java (Yu et al. 2016).

By far the best-known species is *E.yokahamae* which is relatively common in Japan and has a remarkably long ovipositor. Despite its impressive size we present the first molecular data for this species. *Euurobraconyokahamae* has been recorded in the literature as parasitising two species of cerambycid beetles, *Batoceralineolata* Chevrolat, 1852 (Watanabe 1934; repeated in Shenefelt 1978) and of the pupa of *Neocerambyxraddei* Blessig & Solsky, 1872 (syn. *Massicusraddei* (Blessig & Solsky, 1872)) (Kaga et al. 2018; Cao et al. 2020). However, Kaga et al. (2018) strongly infer that the record from *Batocera* is incorrect because the beetle’s ecology makes this unsuitable to be a host. *Euurobraconyokahamae* adults overwinter in wood (e.g., *Quercusserrata* and *Castaneacrenata*). Kaga et al. (2018) noted that oviposition occurs by inserting the ovipositor into cracks in the wood and suggest that it is highly unlikely that they lay eggs directly on the host. The sex ratio is approximately one for specimens extracted from wood prior to emergence but that of individuals collected/observed in the field is markedly female-biased, with males being rare. This might be due to males being short-lived and/or being less conspicuous. In Japan, *E.yokahamae* adults are observed in the fields from late spring to early summer (late April to early June).

Here we describe a new species from West Java (Indonesia), *E.bhaskarai* Quicke sp. nov., and differentiate it from the closely related *E.yokahamae* on the basis of colour pattern, morphology and DNA sequence data for three mitochondrial genes.

## ﻿Material and methods

### ﻿Materials

Terminology follows van Achterberg (1988) except for wing venation nomenclature which follows Sharkey and Wharton (1997); see also figure 2.2 in Quicke (2015) for comparison of wing venation naming systems. Specimens of *E.bhaskarai* sp. nov. were photographed using a Leica M205 C microscope. Images were prepared by image stacking using Leica Application Suite (LAS).

### ﻿Abbreviations

**CUMZ**Entomological Museum, Chulalongkorn University, Bangkok;

**KPM-NK**Kanagawa Prefectural Museum of Natural History, Japan;

**MZB**Museum Zoologicum Bogoriense (Zoological Museum), Indonesian Institute of Sciences, Bogor, Indonesia.

### ﻿Molecular methods

DNA sequences were generated for the barcoding region of cytochrome oxidase c subunit 1 (COI), cytochrome b (cytb), 16S rDNA (16S), elongation factor 1-alpha (EF-1𝛼), and the D2-D3 expansion region of 28S rDNA (28S) for four specimens of *E.bhaskarai* Quicke sp. nov., five specimens of *E.yokahamae* (deposited in KPM-NK), and one each of *E.breviterebrae* (deposited in KPM-NK), and *E.cephalotes* (deposited in CUMZ). An additional COI barcode for *E.breviterebrae* was obtained from GenBank.

A

A combined barcoding and phylogenetic rapid bootstrap tree was generated using the maximum likelihood program RAxML (Stamatakis 2014). Combined sequences from two species of the putatively closely related Afrotropical genus *Archibracon* Saussure, (1890) 1892, (Quicke 1989b) were used to root the tree.

Gene sequences are deposited on GenBank and accession numbers are given in Table 1.

**Table 1.** GenBank accession numbers for newly generated sequences.

Taxon	BOLD process ID	GenBank accession numbers				
		CO1	cytb	16S	28S	EF-1𝛼
*Archibracon* sp.	BBTH2575-21	OM950937	OM950960	OM952149	OM950949	–
*E.bhaskarai* sp. nov.	BBTH2844-21	OM950947	OM950970	OM952159	OM950958	OM950980
*E.bhaskarai* sp. nov.	BBTH2843-21	OM950940	OM950963	OM952152	OM950951	OM950974
*E.bhaskarai* sp. nov.	BBTH2841-21	OM950939	OM950962	OM952151	OM950950	OM950973
*E.bhaskarai* sp. nov.	BBTH2678-21	OM950944	OM950967	OM952156	OM950955	OM950977
*E.bhaskarai* sp. nov.	BBTH2840-21	OM950948	OM950971	OM952160	OM950959	OM950981
*E.breviterebrae*	BBTH2845-21	OM950942	OM950965	OM952154	OM950953	OM950975
*E.cephalotes*	BBTH2676-21	OM950943	OM950966	OM952155	OM950954	OM950976
*E.yokahamae*	BBTH2842-21	OM950938	OM950961	OM952150	–	OM950972
*E.yokahamae*	BBTH2839-21	OM950941	OM950964	OM952153	OM950952	–
*E.yokahamae*	BBTH2677-21	OM950945	OM950968	OM952157	OM950956	OM950978
*E.yokahamae*	BBTH2846-21	OM950946	OM950969	OM952158	OM950957	OM950979

## ﻿Results

### ﻿Molecular analysis

**Figure 1.**
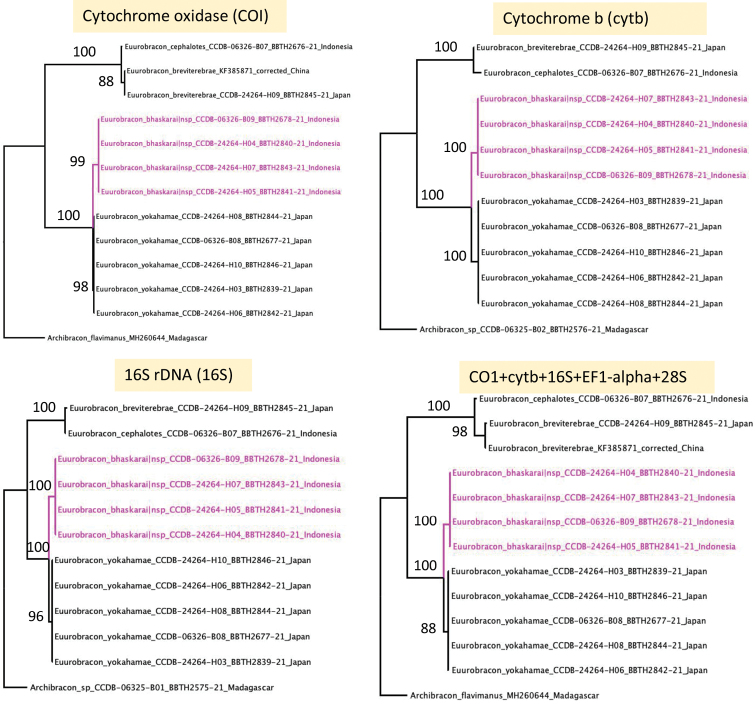
Maximum likelihood bootstrap trees of all available *Euurobracon* sequences rooted with a representative of the putatively closely related Afrotropical genus *Archibracon* Saussure. Individual trees are shown for cytochrome oxidase (COI), cytochrome b (cytb) and 16S rDNA (16S).

No intraspecific variation was present in any of the gene fragments. All genes recovered the phylogeny: ((*bhaskarai* sp. nov. + *yokahamae*) (*cephalotes* + *breviterebrae*)) (Fig. 1). All mitochondrial genes separated *E.bhaskarai* sp. nov. from *E.yokahamae* with high bootstrap support (96–100%). The two species differed by 16 bases in the CO1 gene (2.5%), by 13 bases in the cytb gene (3.37%) and by 10 indels in the 16S gene (2.37%).

The 28S gene and EF-1𝛼 did not differentiate *E.bhaskarai* sp. nov. from *E.yokahamae*, but consistently split *Euurobracon* into two species groups (Fig. 1), the differences in 28S between these groups being entirely indels.

﻿

### ﻿Taxonomy

#### ﻿ Euurobracon

Taxon classificationAnimaliaHymenopteraBraconidae

Ashmead, 1900

DCCA0E6A-6BEC-5E49-AF14-6BC2362BF4F1

Euurobracon Ashmead, 1900: 140. Type species: Braconpenetrator Smith, 1877 not Smith, 1863.
Delmira Cameron, 1900: 87. Type species: Delmiratriplagiata Cameron [Synonymized by Baltazar 1961.]
Exobracon Szépligeti, 1902: 45. Type species: Braconquadriceps Smith, 1861 not Smith, 1858. [Synomymized by Roman 1913.]
Lissobracon Cameron, 1905:103. Type species: Lissobraconforticornis Cameron [Synonymized by Roman 1913.]

##### Diagnosis.

**Figures 2–7.**
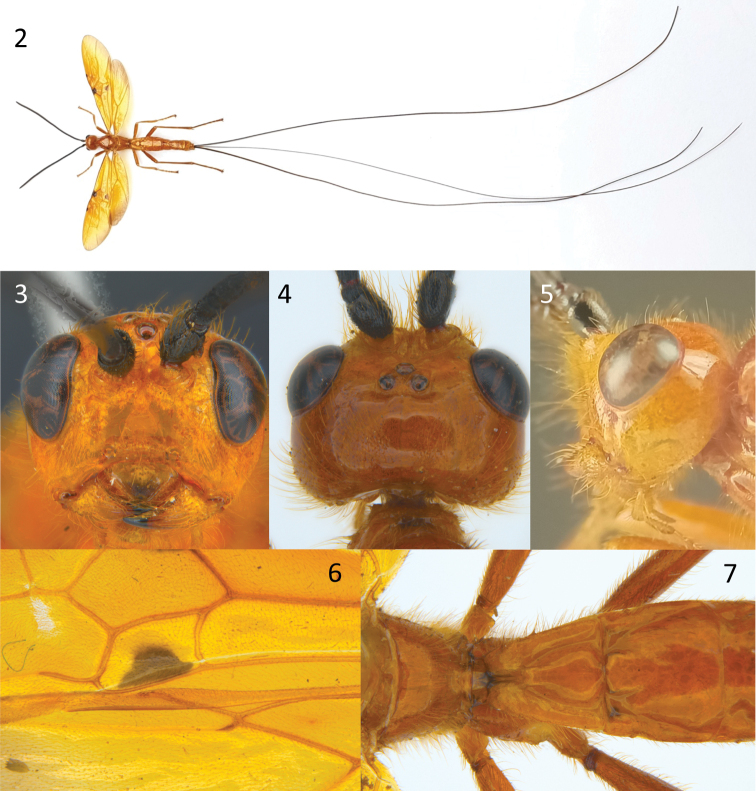
*Euurobraconbhaskarai* Quicke sp. nov, ♀, holotype **2** habitus, dorsal view **3** head, anterior view **4** head, dorsal view **5** head, lateral view **6** medial part of fore and hind wings **7** propodeum and metasomal tergites 1 and 2, dorsal view.

**Figures 8–12.**
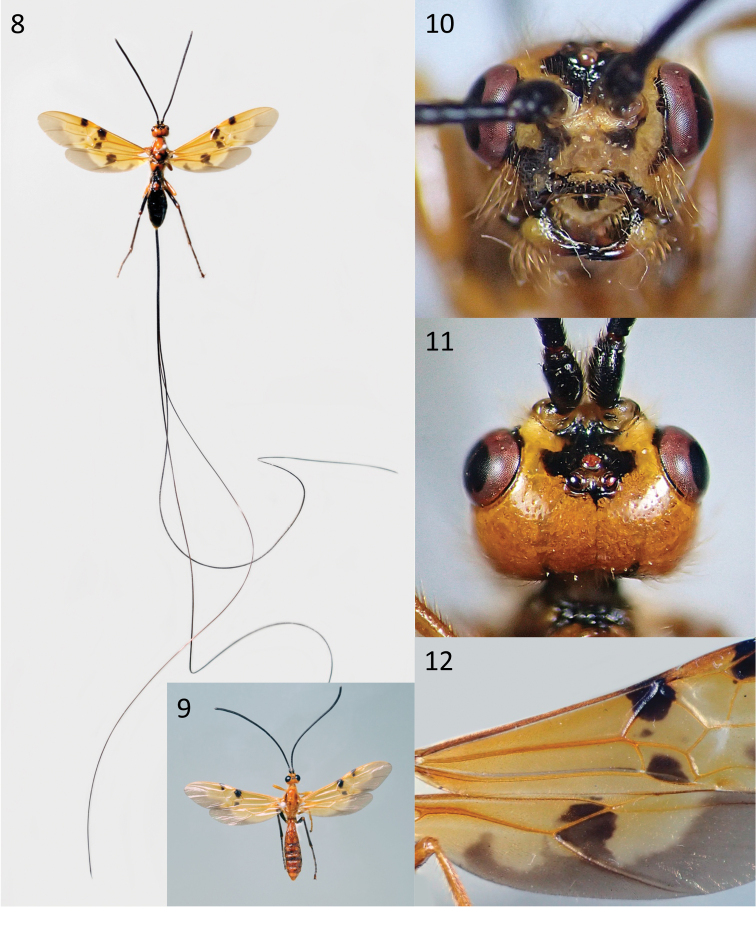
*Euurobraconyokahamae***8** ♀ habitus, dorsal view **9** ♂ habitus, dorsal view **10** ♀ head, anterior view **11** ♀ head, dorsal view **12** ♀ basal two-thirds of fore and hind wings.

Scapus subglobose, shorter ventrally than dorsally in lateral aspect; face flat, clypeus not separated from face by a ridge or carina; propodeum smooth medio-posteriorly (Fig. 7); fore wing vein 3RSa of fore wing 2.5–3.4 × vein 2RS (Figs 2, 8); fore wing vein 1cu-a strongly postfurcal and curved (except *E.interstitialis*), vein 1CUb 3.0–4.6 × 1CUa (Figs 6, 12); hind wing vein 1r-m much longer than SC+R1 (Figs 6, 12); hind wing vein 1r-m short longitudinal (Fig. 12) to shortly transverse (Fig. 6); surroundings of vein cu-a of hind wing setose; metasoma smooth and shiny, first tergite with smooth, polished, convex median area bordered laterally by pair of distinct furrows, with at most weak indication of rounded, dorsal carina anteriorly, without dorsolateral carinae; second metasomal tergite smooth, with at most a weak medio-anterior pair of short converging grooves but without mid-basal triangular area; second suture smooth; fifth and sixth tergites largely exposed and smooth; hypopygium large, reaching or projecting beyond apex of metasoma; ovipositor strongly exserted; dorsal valve of ovipositor with small pre-apical nodus.

The adult rectum is unique among braconids in having a large number (>12) of small rectal pads, compared with four in other genera (Quicke et al. 1999).

#### ﻿ Euurobracon bhaskarai

Taxon classificationAnimaliaHymenopteraBraconidae

Quicke sp. nov.

CC000E75-5F0F-500A-97B1-E7BA1D69DCBF

http://zoobank.org/D6533B79-844D-43DA-B6E3-66CC88BB8C19

Figs 2–7

##### Type material.

***Holotype*** ♀, Indonesia, West Java, nr Mt Halimun, ii.2021, local collector, DNA voucher CCDB-24624-H04 (MZB). ***Paratypes***: 3 ♀, same data as holotype (1 MZB, DNA voucher CCDB-6326-B09; 2 CUMZ, DNA vouchers CCDB-24624-H05, CCDB-24624-H07).

##### Diagnosis.

Body largely orange-yellow; wings largely yellow, fore wing with greyish margin narrowly infuscate, a small dark brown mark at apex of pterostigma, a dark brown patch around the confluence of veins 1RS, 1-M and (RS+M)a, and a brown patch at the posterior part of the 1^st^ subdiscal cell; hind wing vein R (or RSa) interstitial or short transverse; 2^nd^ metasomal tergite without transverse groove at approximately midlength; ovipositor more than 4 × longer than body. In addition, apex of hind wing basal cell with a small elongate sclerome in the membrane at approximately midlength of 1r-m.

The new species is morphologically very similar *E.yokahamae*, the only other predominantly yellow species with very long ovipositor. Nearly all specimens of *E.yokahamae* have hind wing vein R longitudinal (i.e., vein RSa arising from R distal to 1r-m); very rarely it is interstitial. In *E.bhaskarai* sp. nov. vein RSa is short but distinctly transverse or occasionally interstitial. The most conspicuous difference is in the extent of the dark markings of the fore wing. There is some variation in wing colour pattern in female *E.yokahamae* and this was illustrated by Sonan (1932), but this does not include restriction of the fore wing grey pattern to a faint narrow margin with just three small brown spots as in the new species. In addition, in the distal part of hind wing basal cell there is a small thickening of the cell membrane creating a tiny sclerome which is absent in *E.yokahamae*. The antennal flagellum of the four available specimens of the new species is parallel-sided whereas in *E.yokahamae* it is distinctly widened distally.

##### Description.

Length of body 19.5–23.5 mm, of fore wing 18.7–20.0 mm, of antenna 16.6–18.0 mm and of ovipositor, 97–123 mm. ***Head*.** Antenna with 70–71 flagellomeres, more or less parallel-sided. Terminal flagellomere tapering progressively to a point and distinctly acuminate, approximately 1.5 × longer than basally wide. Median flagellomeres transverse, 1.5 × wider than long. Length of first flagellomere: second flagellomere: third flagellomere = 1.45: 1.1; 1.0, the latter being more or less quadrate. Width of head: width of face: height of eye (measured at level of antennal socket) = 2.5: 1.45: 1.0. Dorsal half of clypeus densely long setose. Face densely long setose except for a small median triangular area above the clypeus. Inter-tentorial distance 1.25 × tentorio-ocular distance. Frons sparsely setose. Head widest behind eyes; length of head behind eye 1.1 × length of eye in dorsal view. Malar space 0.9 × longer than basal width of mandible. Minimum length of malar space located at above inner articulation of mandible. Shortest length of mandible 1.2 × longer than basal width of mandible. ***Mesosoma*.** Mesosoma 1.75 × longer than high. Middle lobe of mesoscutum often largely moderately densely setose laterally. Notauli present anteriorly only. Anterior margin of propodeum without a deep medial emargination. Propodeal spiracle elongate, ca 2.0 × longer than maximum width. ***Wings*.** Fore wing vein 1cu-a far postfurcal, vein 1CUb 3.3 × 1CUa. Forewing vein 2CUa only weakly and gradually expanded posteriorly. Vein (RS+M)a 1.0–1.1 × length of 1-M. Forewing vein m-cu straight, 2.0 × longer than (RS+M)b. Lengths of fore wing veins r-rs: 3RSa: 3RSb = 1.0: 5.5: 6.0. Lengths of fore wing veins 2RS: 3RSa: rs-m = 1.0: 2.75: 1.1. Hindwing vein 1r-m approximately 1.3 × longer than R1 before it reaches wing margin hind wing vein R marginally longitudinal, interstitial or marginally transverse (i.e., with very short vein rs-m). ***Legs*.** Length of fore femur: tibia: tarsus = 1.0: 1.0: 1.25. Anterolateral aspect of fore tibia more or less densely clothed with slightly thickened setae. Fore basitarsus 4.3 × longer than maximally deep. Length of hind femur: tibia: basitarsus = 1.0: 1.4: 1.2. Hind femur 6.0 × longer than maximum depth in lateral view. Hind tibia 12.5 × longer than maximum depth in lateral view. Hind basitarsus 8.3 × longer than deep. ***Metasoma*.** First tergite 1.2 × longer than maximally wide. Second tergite smooth, 1.2–1.35 × wider than long, without any trace of median transverse groove or furrow. Second + third metasomal tergites 1.3–1.4 × longer than maximally wide. Ovipositor long, 5.2–6.2 × forewing, 5.0–5.3 × longer than body. ***Coloration*.** Antenna black. Head, including stemmaticum, and body uniformly ferruginous-yellow (orange-yellow), usually with few black marks as follows: posterior margin of propodeum, medio-anterior of tergite 1, anterolateral part and longitudinal sublateral grooves of tergite 2, anterolateral part of tergite 3, posterior margins of tergites 3–5. Wings largely yellow, narrowly weakly infuscate distally and postero-distally, with dark brown marks at apex of pterostigma, around junction of veins 1RS, 1-M and (RS+M)a but excluding parastigma, and posterior part of first subdiscal cell, membrane. Fore and mid legs ferruginous-yellow except apex of hind tibia and basal three hind tarsomeres which are piceous. Ovipositor sheaths black.

**Male.** Unknown.

##### Etymology.

Named after Mr Edy Bhaskara, friend of the first author, who lives on the island where the new species was collected.

##### Biology.

Unknown.

#### ﻿ Euurobracon yokahamae

Taxon classificationAnimaliaHymenopteraBraconidae

(Dalla Torre, 1898)

1F126D2B-A450-5D20-9862-C5B8785086A9

Figs 8–12

##### Material examined.

Japan, Honshu, KPM-NK62083, ♀, Kanagawa Pref., Yokohama City, Aoba-Ku, Jike, 9.v.2017; KPM-NK62090, ♀, Kanagawa Pref., Yokohama City, Midori-Ku, Mihomachi, 11.v.2017; KPM-NK55278, ♀, Kanagawa Pref., Sagamihara City, Midori-Ku, Magino, 20.v.2019, H. Karube leg.; KPM-NK51571, ♀, Kanagawa Pref., Aikawa Town, Mimase, 2.v.2017, H. Karube leg.; KPM-NK62092, ♀, Kanagawa Pref., Aikawa Town, Sumida, 12.v.2017, H. Fujita leg.; KPM-NK51570, ♀, Kanagawa Pref., Hiratsuka C., Kisawa, 1.v.2017, H. Karube leg.; KPM-NK47713, ♀, Kanagawa Pref., Oiso Town, Nishikoiso, 12.v.2017; KPM-NK55279, ♀, Kanagawa Pref., Nakai Town, Zoushiki, 7.v.2019, K. Watanabe leg.; KPM-NK62086, ♀, Kanagawa Pref., Hadano City, Horikawa, 6.i.2016 (from dead tree), K. Suzuki leg.; KPM-NK47711, ♀, Kanagawa Pref., Hadano City, Mt. Koubou-yama, 19.v.2017, R. Kaga leg.; KPM-NK69393, ♂, same locality, 30.v.2018 (host coll.), 13.vii.2018 (em.), R. Kaga et al. leg.; KPM-NK69395, 69389, 69405, 1 ♀ & 2 ♂, Kanagawa Pref., Hadano City, Mt. Koubou-yama, 30.v.2018 (host coll.), 14. VII. 2018 (em.), R. Kaga et al. leg.; KPM-NK69402, 69403, 2 ♂, Kanagawa Pref., Hadano City, Mt. Koubou-yama, 30.v.2018 (host coll.), 18.vii.2018 (em.), R. Kaga et al. leg.; KPM-NK62085, ♀, Kanagawa Pref., Ooi Town, Yamada, 11.v.2017, H. Karube leg.; KPM-NK62379, ♂, Kanagawa Pref., Minamiashigara City, Iwahara, 7.x.2015 (from dead tree), K. Suzuki leg.; KPM-NK47708, ♀, Kanagawa Pref., Minamiashigara City, Tsukahara, 11.v.2017, H. Karube leg.; KPM-NK62089, ♀, Shizuoka Pref., Fukuroi City, Tsurugaike, 5.v.2017, H. Karube leg.; KPM-NK55276, ♀, Yamanashi Pref., Nirasaki City, Hosaka Town, Mitsuzawa, 11.v.2018, H. Fujita leg.; KPM-NK55277, ♀, Gifu Pref., Ena City, Okasezawa, 11.v.2019, H. Karube leg.

##### Description.

We update the description of this species proposed by Quicke (1989a) based on examination of the above material, including males.

**Females**: Length of body 14.5–21.5 mm, of forewing 15.5–23.0 mm, and of ovipositor 85–204 mm. ***Head*.** Antenna with 65–77 flagellomeres (the number of articles is approximately proportional to the body length), distinctly widening distally to approximately 1.2 × width near base. Terminal flagellomere tapering progressively to a point and distinctly acuminate, approximately 1.5 × longer than basally wide. Median flagellomeres transverse, 1.5 × wider than long. Lengths of first flagellomere: second flagellomere: third flagellomere = 2.0: 1.65: 1.8–2.0. First flagellomere more or less parallel-sided except for slight basal flare, the latter being more or less quadrate. Head widest across eyes, 0.7 and 0.7–1.1 × longer than maximum width of eye and of gena in dorsal view, respectively. Width of head: width of face (measured at height of antennal socket): height of eye = 2.7: 1.6: 1.0. Length of head behind eye 1.0–1.5 × length of eye in dorsal view. Maximum width of gena 1.0–1.4 × longer than maximum width of eye in lateral view. Dorsal half of clypeus densely long setose. Face densely long setose except for a small median triangular area immediately above the clypeus. Malar space 0.8–1.15 × longer than basal width of mandible. Minimum length of malar space located at above inner articulation of mandible. Shortest length of mandible 1.1–1.3 × longer than basal width of mandible. Shortest distance between eyes 0.55–0.6 × longer than maximum width of head in frontal view. Frons largely densely short setose except for median area sparsely setose. POL: diameter of posterior ocellus: shortest distance between posterior ocellus and eye = 0.45–0.9: 1.0: 2.5–2.7. Occiput moderately densely setose. ***Mesosoma*.** Mesosoma 1.6–1.7 × longer than high. Middle lobe of mesoscutum often largely moderately densely setose. Notauli present, ending posteriorly near centre of mesoscutum. Anterior margin of propodeum without a deep medial emargination. Propodeal spiracle elongate, ca 2.0 × longer than maximum wide. ***Wings*.** Fore wing vein 1cu-a far postfurcal, vein 1CUb 3.0–4.6 × 1CUa vein. Forewing vein 2CUa usually only weakly and gradually expanded posteriorly. Lengths of fore wing veins r-rs: 3RSa: 3RSb = 1.0: 4.3–4.8: 6.1–6.5. Vein (RS+M)a 1.0–1.1 × length of 1-M. Forewing vein m-cu straight, 1.6–2.2 × longer than (RS+M)b. Lengths of forewing veins 2RS: 3RSa: rs-m = 2.0: 2.1–2.3: 0.9–0.95. Hindwing vein 1r-m approximately 1.55 × longer than R1 before it reaches wing margin. Hindwing vein R usually longitudinal, rarely interstitial. ***Legs*.** Lengths of fore femur: tibia: tarsus = 1.0: 1.0–1.15: 1.1–1.12. Anterolateral aspect of fore tibia more or less densely clothed with slightly thickened setae. Fore basitarsus 4.3–4.5 × longer than maximally deep. Lengths of hind femur: tibia: basitarsus = 1.0: 1.5: 1.5–1.75. Hind femur 5.0–5.8 × longer than maximum depth in lateral view. Hind tibia 9.5–11.0 × longer than maximum depth in lateral view. Hind basitarsus 7.7 × longer than deep. ***Metasoma*.** First metasomal tergite 0.98–1.24 × longer than maximally wide (generally shorter in length for larger specimens); dorsal carinae relatively close together, broadly rounded ridges rather than lamelliform carinae; raised median area with or without a mid-longitudinal groove. Second metasomal tergite largely glabrous 1.25–1.43 × wider than long, without a distinct transverse median groove on either side of the midline, with a pair of sublateral oblique furrows. Third metasomal tergite with distinct anterolateral areas, without a pair of sub-medial transverse grooves or pits. Second + third metasomal tergites 1.1–1.3 × longer than maximally wide. Ovipositor long, 5.55–9.3 × forewing length [5.0–14.0 in Quicke (1989a)] though generally between 6.0 and 9.0 × fore wing]; 5.85–9.5 × longer than body. Apex of lower valve of ovipositor with five teeth. Approximately distal 0.1 of lower valve of ovipositor with rough surface laterally (and also ventrally except for teeth). ***Coloration*.** The additional materials completely agree with the character states of coloration proposed by Quicke (1989a) which is reproduced below. Antenna and ovipositor sheath black. Body usually largely or entirely ferruginous-yellow (somewhat paler in the Indian specimens), sometimes with piceous markings especially on the metasomal tergites, propodeum, metanotum, mesopleuron and propodeum. Fore and middle legs ferruginous-yellow, hind legs usually black or dark piceous but entirely yellow in specimens from India, Laos and Thailand. Wings yellow with a somewhat variable brown pattern (Sonan, 1932), the distinctive features being: a dark mark at the parastigma and at the apex of the pterostigma of the forewing; usually a dark mark in the first subdiscal cell of the forewing; a pale grey-brown at the apex of the forewing, extending and darkening slightly along the postero-distal part of the wing margin; hindwing with a grey-brown apical region which extends along the posterior wing margin and is produced into the base of the submarginal cell and again into the discal+subdiscal cells.

**Males**: Similar to female. Length of first flagellomere: second flagellomere: third flagellomere = 2.0: 1.1–1.3: 1.4–1.55. Head 1.1–1.15 × longer than maximum width of gena in dorsal view. Eye relatively larger than in female, maximum width of gena 0.5–0.6 × longer than maximum width of eye in lateral view. Shortest distance between eyes 0.25–0.3 × longer than maximum width of head in frontal view. Face slightly narrower than female, 0.43–0.7 × longer than maximum width. Ocelli larger than female. POL distinctly longer than shortest distance between posterior ocellus and eye. POL: diameter of posterior ocellus: shortest distance between posterior ocellus and eye = 0.5–0.6: 1.0: 0.3–0.5. Malar space (minimum length) 2.0 × longer than basal width of mandible. Minimum length of malar space located at above outer articulation of mandible. Length of hind femur: tibia: basitarsus = 1.0: 1.55–1.8: 1.35–1.6. First metasomal tergite slenderer than female, 1.25–1.4 × longer than maximum width. Second and third metasomal tergites usually with slight transverse depression. ***Male genitalia*.** Basal ring V-shaped, its dorsal part narrow and linear. Digitus large and triangular, with three minute tubercles at apex. Apex of paramere not projecting beyond apex of aedeagus, densely setose. Dorsal surface of aedeagus largely flat subapically. Ventral side of aedeagus with lamella-like expansion. Described in detail and illustrated by SEM in Quicke (1988).

##### Distribution.

Japan, Korea, China, Taiwan, Laos, Thailand and India (Yu et al. 2016).

##### Biology.

See Introduction.

##### Remarks.

A set of eight figures showing the range of variation of forewing markings found in the East Palaearctic population was provided by Sonan (1932). Quicke (1989a) classified this species into its own, monotypic, *E.yokahamae* species group which is recognizable from all other *Euurobracon* spp. by its interstitial or longitudinal hindwing vein 2-SC+R in combination with predominantly yellow or red-yellow coloration.

## ﻿Discussion

In general, even among parasitoid wasps, large and brightly coloured species, have often already been described, and indeed, not uncommonly have one or more synonyms (Jones et al. 2009, 2011; Quicke 2012). Most of the undescribed diversity occurs among the smaller and less spectacular groups. Nevertheless, in South-east Asia, and adjacent southern China, many such new species are being described. Members of the genus *Euurobracon* are, despite their large body size and often impressive ovipositors, almost certainly vastly under-recorded in South-east Asia.

## Supplementary Material

XML Treatment for Euurobracon

XML Treatment for Euurobracon bhaskarai

XML Treatment for Euurobracon yokahamae
